# ΕGFR/ERβ-Mediated Cell Morphology and Invasion Capacity Are Associated with Matrix Culture Substrates in Breast Cancer

**DOI:** 10.3390/cells9102256

**Published:** 2020-10-08

**Authors:** Konstantina Kyriakopoulou, Eirini Riti, Zoi Piperigkou, Konstantina Koutroumanou Sarri, Heba Bassiony, Marco Franchi, Nikos K. Karamanos

**Affiliations:** 1 Biochemistry, Biochemical Analysis & Matrix Pathobiology Research Group, Laboratory of Biochemistry, Department of Chemistry, University of Patras, 26504 Patras, Greece; k.kyriakopoulou@upnet.gr (K.K.); eirhnh.rhti@gmail.com (E.R.); zoipip@upatras.gr (Z.P.); nantiakout@icloud.com (K.K.S.); 2 Department of Zoology, Faculty of Science, Cairo University, Cairo 11865, Egypt; heba_b683@hotmail.com; 3 Department for Life Quality Study, University of Bologna, 47921 Rimini, Italy

**Keywords:** breast cancer, estrogen receptor beta, EGFR, tumor microenvironment, filopodia, extravesicles, tunneling nanotubes, scanning electron microscopy

## Abstract

Breast cancer accounts for almost one in four cancer diagnoses in women. Studies in breast cancer patients have identified several molecular markers, indicators of aggressiveness, which help toward more individual therapeutic approaches. In triple-negative breast cancer (TNBC), epidermal growth factor receptor (EGFR) overexpression is associated with increased metastatic potential and worst survival rates. Specifically, abnormal EGFR activation leads to altered matrix metalloproteinases’ (MMPs) expression and, hence, extracellular matrix (ECM) degradation, resulting in induced migration and invasion. The use of matrix substrates for cell culture gives the opportunity to mimic the natural growth conditions of the cells and their microenvironment, as well as cell–cell and cell–matrix interactions. The aim of this study was to evaluate the impact of EGFR inhibition, estrogen receptor beta (ERβ) and different matrix substrates [type I collagen and fibronectin (FN)] on the functional properties, expression of MMPs and cell morphology of ERβ-positive TNBC cells and shERβ ones. Our results highlight EGFR as a crucial regulator of the expression and activity levels of MMPs, while ERβ emerges as a mediator of MMP7 and MT1-MMP expression. In addition, the EGFR/ERβ axis impacts the adhesion and invasion potential of breast cancer cells on collagen type I. Images obtained by scanning electron microscope (SEM) from cultures on the different matrix substrates revealed novel observations regarding various structures of breast cancer cells (filopodia, extravesicles, tunneling nanotubes, etc.). Moreover, the significant contribution of EGFR and ERβ in the morphological characteristics of these cells is also demonstrated, hence highlighting the possibility of dual pharmacological targeting.

## 1. Introduction

Breast cancer affects more than two million women worldwide every year [1], highlighting the importance of improved diagnosis, better management and the integration of personalized treatment [2]. Advances in diagnostic methods (e.g., mammography screening, analysis of serum tumor markers and/or circulating tumor cells, imaging methods, etc.) significantly helped in reducing mortality rates [3,4,5], though these methods prove to be inept when applied to early detection of metastasis. Metastasis has proven to be the single most significant factor that contributes to the high mortality rates of breast cancer. Specifically, recent data show that metastasis is responsible for two-thirds of breast-cancer-related deaths [6]. Metastasis is a complex process that involves extracellular matrix (ECM) degradation, cell intravasation in the lymphatic vessels and the bloodstream, extravasation in the distant organs/tissue and, finally, the establishment of secondary tumors [7]. Consequently, further understanding of the biochemical and biomechanical mechanisms, in particular epithelial-to-mesenchymal transition (EMT) and interactions of matrix substrates with integrins and other cell-surface receptors, that drive the interplay between cancer cells and tumor stroma during cancer progression will be of strategic relevance toward cancer prevention and drug treatment development.

The interactions that occur between cancer cells and tumor microenvironment (TME) are crucial for determining the metastatic potential of the cells. Growth factors and receptor tyrosine kinases (RTKs) are among the essential molecules that drive EMT and therefore facilitate metastasis. In triple-negative breast cancer (TNBC), the most aggressive type of the disease with the worst prognosis mainly due to relapse, epidermal growth factor receptor (*EGFR*) is significantly overexpressed [8]. The high expression and subsequent activation of EGFR strongly stimulates its downstream signaling pathways, hence the transcription of target genes associated with various cell processes such as invasion, proliferation and EMT [9,10]. More specifically, phosphoinositide 3-kinase (PI3K)/AKT, Ras/Raf/mitogen-activated protein kinase (MAPK), janus kinase (JAK)/signal transducers and activators of transcription (STAT) and c-Src tyrosine kinase pathways are all induced following EGFR activation [11,12]. During EMT, cells are subjected to significant biochemical changes that result in morphological changes; polygonal and cobble-stone-shaped cells with apical-to-basal polarity and tight cell–cell junctions turn to spindle-like cells with mesenchymal traits, namely front-to-back polarity, reorganized cytoskeleton and increased motility [13]. This transition is accompanied by elevated migratory potential, invasiveness and altered ECM composition [14].

ECM, one of the most widely studied areas in TME of the last decade, is a complex, vast network of macromolecules that serves both as a structural support for the cells and as a signal transducer that regulates cellular processes such as migration and invasion [15]. Matrix metalloproteinases (MMPs), the main ECM remodeling enzymes, have a physiological role in normal mammary gland branching morphogenesis, angiogenesis and wound-healing, among others [16]. When abnormally expressed, they can cause alterations of the biomechanical properties of ECM and guide invasion and metastasis, hence cancer progression [17]. Notably, MMPs -2, -7, -9 and -14 (membrane-type 1 MMP/MT1-MMP) are among those overexpressed in breast cancer, and various studies in breast cancer patients have identified them as prognostic markers for breast cancer progression, as well as therapeutic targets [16,18].

Intriguingly, cancer cells undergo significant molecular changes that, along with the dynamic interplay with the surrounding stroma, governed by the tumor microenvironment, drive cancer progression and may govern future therapeutic actions [19]. Interactions of cancer cells with matrix macromolecules of the tumor microenvironment play key roles in cancer development, such as EMT, angiogenesis and invasion, thus promoting tumor growth and metastasis [20]. A critical initial step toward this process is the formation of invadopodia. Invadopodia are rich in F-actin membrane protrusions that induce ECM degradation through MMP deposition, thus enabling the penetration of cancer cells in tumor stroma [21,22]. Other structures responsible for increased motility and migratory potential include lamellipodia and filopodia. Lamellipodia are highly dynamic membrane protrusions in the front region of migrating cells [23]. Filopodia are thin “finger-like” membrane projections made of parallel actin filaments and as with lamellipodia, they appear at the leading edge of the migrating cells [24,25]. Their function is to command the direction of the migratory cells in physiological processes like embryonic development, wound-healing, and ECM adhesion, as well as in cancer, where they also contribute to cancer cell invasion [26,27]. Moreover, a newly emerging way of cell communication, tunneling nanotubes (TNTs), is attracting attention due to their ability to provide cell–cell connection and transfer various components [28]. Specifically, TNTs facilitate extracellular vesicles (EVs) secretion that is not only known to promote invadopodia formation but also to increase the invasive capacity independently of growth factor stimulation [29,30]. Thus, TNTs can advance embryonic development but also cancer progression and chemoresistance [28,31].

Collagens and FN are two of the main structural proteins of the ECM. They play crucial roles in cell–matrix adhesion and signaling. Collagen I is a prominent fibrillar protein present in connective tissues, and also one of the most widely used artificial matrices [32,33]. It has a pivotal role in tumorigenesis, as it is not only responsible for the mechanical properties (stiffness, contractility, adhesion, etc.) of the ECM, but it can also interact with other matrix effectors, thus influencing cell behavior [34]. FN, on the other hand, is ubiquitously expressed in tissues and capable to form suprastructured fibril networks. FN contributes to the development of tumor blood vessel networks, hence advancing tumor progression [15]. Moreover, its interactions with ECM effectors facilitate the activation of various signaling pathways linked with EMT, invasion and metastasis [35].

Recent studies in TNBC implicate ERβ as an effector of matrix macromolecules, as well as cellular signaling [36]. Additionally, previously published data have established a correlation between estrogen receptor beta (ERβ) expression and the development of distinct morphological characteristics like long filopodia and TNTs in breast cancer cells when cultured on matrix substrates [37]. In this paper, we address the role of EGFR in the functional cell properties, such as adhesion on collagen type I, invasion capacity and metastatic potential, as well as the expression of key matrix macromolecules related with cancer progression. In addition, we highlight the role of EGFR in determining cell morphology in cultures on major cell–matrix mediators (collagen I and FN substrates). SEM images of TNBC cells of different ERβ status offer a unique insight on the influence of EGFR inhibition, as well as exogenous addition of 17β-estradiol (E2) in cell shape, development of cytoplasmic protrusions and production of EVs. These novel data emphasize the functional role of EGFR signaling in modulating cell morphology in MDA-MB-231 (ERβ-positive) and shERβ MDA-MB-231 (ERβ-suppressed) triple-negative breast cancer cells. Moreover, our data suggest the vital role of different matrix substrates in modifying breast cancer cell morphology and the formation of cell protrusions and surface structures, as an underlying mechanism contributing to better understanding breast cancer progression.

## 2. Materials and Methods

### 2.1. Chemicals and Reagents

Dulbecco’s modified Eagle medium (DMEM), fetal bovine serum (FBS), PBS, trypsin- Na_2_EDTA, L-glutamine, penicillin and streptomycin were all obtained from Biosera LTD (Courta-boeuf CEDEX, France). FN was supplied by BD Biosciences (NJ, USA). Type I collagen was supplied by Sigma-Aldrich (Steinheim, Germany). E2 and allosteric inhibitor of EGFR, AG1478, were supplied by Sigma Chemical Co. (St. Louis, MO, USA). All other chemicals used were of the best commercially available grade.

### 2.2. Cell Culture

MDA-MB-231 (high metastatic potential, ERα-negative, ERβ-positive) breast cancer cell line was obtained from the American Type Culture Collection (ATCC, Baltimore, MD, USA) and routinely cultured as monolayers at 37 °C in a humidified atmosphere of 5% (*v*/*v*) CO_2_ and 95% air. shERβ ΜDA-MB-231 (ERβ-suppressed cells) were previously described [36]. MDA-MB-231-aggressive, mesenchymal-like cell line is routinely utilized in studies of the invasion and metastatic potential of breast cancer cells. To further investigate the role of EGFR/ERβ axis, shERβ MDA-MB-231 cells (70% ERβ suppression compared to MDA-MB-231 cells) were also used. ERβ suppression results in a partial mesenchymal-to-epithelial transition (MET), thus a less aggressive cell line with epithelial-like morphological and molecular characteristics. Breast cancer cells were cultured in DMEM supplemented with 10% (*v*/*v*) FBS, 1% L-glutamine and 1% penicillin/streptomycin. Puromycin dihydrochloride (Sigma-Aldrich, Steinheim, Germany) (0.8 μg/mL) was included in the cultures of shERβ ΜDA-MB-231 cells. Cells were harvested by trypsinization with 0.05% (*w*/*v*) trypsin in PBS containing 0.02% (*w*/*v*) Na_2_EDTA. All experiments were conducted in serum-free conditions. To better mimic the ECM and the tumor microenvironment both MDA-MB-231 and shERβ MDA-MB-231 breast cancer cells were seeded in 3D cultures prepared on isopore membrane filters, with a pore size of 8.0 μm (Millipore, Milan, Italy), covered by collagen type I (200 and 3000 µg/mL) or FN (130 µg/mL). Similarly, 3D Millipore cultures were performed as described above prior to AG1478 (2 μM) and/or E2 (10 nM) treatment for 24 h. It should be noted that, in the mix group, AG1478 (2 μM) treatment for 30 min was conducted prior to E2 (10 nM) addition. AG1478 is a widely used allosteric inhibitor of EGFR and has been shown to be effective even in 1 μM and up to 10 μM. To avoid inhibition of other RTKs (Her2 and PDGFR), a protocol based on previous studies in our lab was established where we used 2 μM of AG1478 and that was effective in both MDA-MB-231 and shERβ MDA-MB-231 cells. Ε2 optimal concentration was determined by IC50 assay previously performed in our lab.

### 2.3. RNA Isolation, Reverse Transcription and Real-Time qPCR Analysis

Breast cancer cells were grown in serum-containing medium up to 70–80% confluence. Cells were serum starved for 16 h. Then, AG1478 (2 μM) and/or E2 (10 nM) were added according to the experimental plan in serum-free culture medium for 24 h. It should be noted that, when both agents were added, AG1478 (2 μM) treatment for 30 min was conducted prior to E2 (10 nM) addition. Total RNA was isolated from cells, using NucleoSpin^®^ RNA II Kit (Macherey-Nagel, Duren, Germany). The amount of isolated RNA was quantified by measuring its absorbance at 260 nm. Moreover, the 260/280 and 260/230 ratios of all the RNA extracts were evaluated to ensure RNA purity. The value of all the 260/280 ratios was ~2.0, and all the 260/230 ratios were between 2.0 and 2.2, which is considered as generally accepted. Total RNA was reverse-transcribed, using thePrimeScript™1st strand cDNA synthesis kit perfect real time (Takara Bio Inc., Göteborg, Sweden) and KAPA Taq ReadyMix DNA Polymerase (KAPA BIOSYSTEMS, Wilmington, MA, USA). Real-time PCR analysis was conducted in 20 μL reaction mixture, according to manufacturer’s instructions. The amplification was performed utilizing Rotor Gene Q (Qiagen, Germantown, MD, USA). All reactions were performed in triplicates, and a standard curve was always included for each pair of primers for assay validation. In addition, a melting curve analysis was always performed for detecting the SYBR Green-based objective amplicon. If more than one peak was detectable, the conditions were optimized. To provide quantification, the point of product accumulation in the early logarithmic phase of the amplification plot was defined by assigning a fluorescence threshold above the background, defined as the threshold cycle (Ct) number. Relative expression of different gene transcripts was calculated by the ΔΔCt method. Τhe Ct of any gene of interest was normalized to the Ct of the normalizer (ACTB). Fold changes (arbitrary units) were determined as 2^−ΔΔCt^. Genes of interest and utilized primers are listed in Table 1.

To cross-check the primers’ properties and their quality, we used two different websites: https://blast.ncbi.nlm.nih.gov/Blast.cgi?PROGRAM=blastn&PAGE_TYPE=BlastSearch&LINK_LOC=blasthome and https://www.idtdna.com/pages/tools/oligoanalyzer. The selection was based on GC content, *E* value (<0.1), self-dimer and hetero-dimer tendencies, query cover (100%) and base pair (bp) size (up to 120 bp).

### 2.4. Cell Invasion Assay

The invasive potential of MDA-MB-231 and shERβ MDA-MB-231 breast cancer cells was evaluated by using collagen type I invasion assay, as described in a previous study [38]. In brief, the collagen type I solution with final concentration of 1 mg/mL was prepared by mixing the precooled components: 4 volumes collagen type I (stock concentration 3 mg/mL), 5 volumes of CMF-HBSS, 1 volume of MEM (10×), 1 volume of 0.25 M NaHCO_3_, 2.65 volumes of standard medium and 0.3 volumes of 1 M NaOH. The solution was gently mixed and added to one well of 12-well plate, spread homogeneously and let gelify in a humidified atmosphere of 10% CO_2_, at 37 °C, for at least 1 h. MDA-MB-231 breast cancer cells were previously cultured in the absence of serum for 16 h and then seeded at a density of 6 × 10^4^ cells per well on top of collagen I type gels. Cells were incubated in a humidified atmosphere of 10% CO_2_, at 37 °C, and, after 24 h of treatment, digital images with objective 10× were obtained. The evaluation of cell invasion was conducted according to the experimental protocol of DeWever et al. [38].

### 2.5. Cell Adhesion Assay

In order to evaluate the adhesion potential of MDA-MB-231 breast cancer cells, the following adhesion protocol was conducted, as it was described by previous works [39,40]. Briefly, 96-well plate was coated with collagen type I solution (40 μg/mL) and kept at 4 °C. After 12 h, the solution was removed, and the plate was air-dried; 3% BSA in PBS solution was added in each well, for 30 min, to block non-specific adsorption. Then the solution was removed, and the plate was washed with PBS and air-dried. Cells treated for 24 h prior to the adhesion assay were detached with PBS-EDTA 1× and resuspended in serum-free medium with 0.1% BSA. and seeded at a density of 2 × 10^4^ cells/well. Cells were incubated for 30 min, to allow adhesion to the surface. Non-adherent cells were removed with serum free medium, and then cells were incubated with medium supplemented with 10% FBS for 2–3 h for recovery. After the incubation period, Premix WST-1 (water-soluble tetra-zolium salt) Cell Proliferation Assay System (Takara Bio Inc., Göteborg, Sweden) was added at a ratio 1:10, and the absorbance at 450 nm was measured (reference wavelength at 650 nm).

### 2.6. Gelatin Zymography

Breast cancer cells were grown in serum-containing medium up to 70–80% confluence. Cells were serum-starved for 16 h. Then the treatments were added according to the experimental plan in serum-free culture medium for 16 h. Gelatin zymography was performed essentially as described [41]. Serum-free conditioned media containing equal amounts of protein were subjected to SDS-PAGE in 10% poly-acrylamide gels, containing 0.1% (*w*/*v*) gelatin. After electrophoresis, the gels were washed with 2.5% Triton X-100, to remove SDS, and then incubated at 37 °C, for 20 h, in 10 mM Tris–HCl, pH 7.5, containing 150 mM NaCl and 5 mM CaCl_2_. Gels were stained for 30 min in 0.25% (*w*/*v*) Coomassie brilliant blue R-250 in 43% (*v*/*v*) methanol/7% (*v*/*v*) acetic acid and destained in 40% (*v*/*v*) methanol/7% (*v*/*v*) acetic acid. Areas of protease activity were detected as clear bands against the blue gelatin background.

### 2.7. SEM Imaging

Breast cancer cells seeded in 3D cultures were fixed in a Karnovsky’s solution for 30 min at 4 °C. The intact Millipore filters covered by different substrates with adhered cells were rinsed three times with 0.1% cacodylate buffer, dehydrated with increasing concentrations of ethanol and finally dehydrated with hexamethyldisilazane (Sigma-Aldrich, Steinheim, Germany) for 15 min. All specimens were mounted on appropriate stubs and coated with a 5 nm palladium gold film (Emitech 550 sputter-coater), to be observed under a SEM (Philips 515, Eindhoven, The Netherlands) operating in secondary-electron mode.

## 3. Results

### 3.1. EGFR Regulates the Expression of Matrix Macromolecules in Triple-Negative Breast Cancer Cells

The well-described crucial role of EGFR in the advancement of breast cancer prompted us to investigate the effects of EGFR inhibition in TNBC cells with different ERβ status (MDA-MB-231 (ERβ-positive) and shERβ MDA-MB-231 (ERβ-suppressed)) in the expression of matrix molecules implicated in breast cancer progression, such as MMP2, -9, -7 and -14 (MT1-MMP).

**Figure 1:**
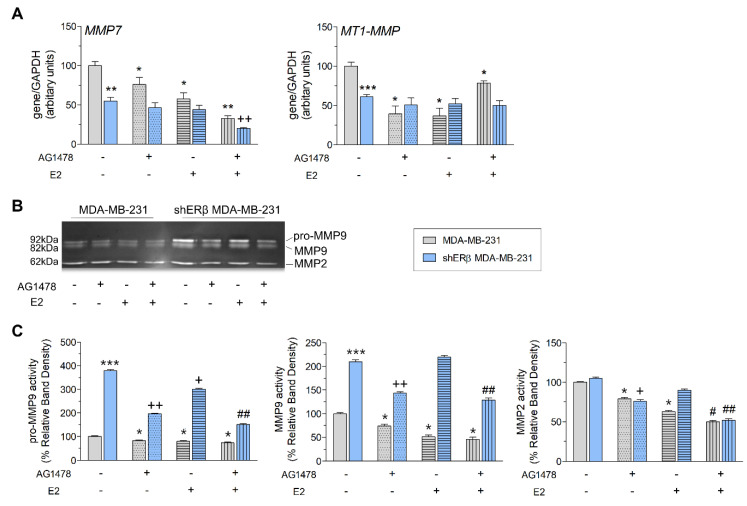
EGFR inhibition affects the expression and activity levels of proteases in MDA-MB-231 and shERβ MDA-MB-231 breast cancer cells. (**A**) Quantitative RT-PCR analysis of *MMP7* and *MT1-MMP* mRNA levels after 24 h without and with treatments (AG1478, E2 and mix). (**B**,**C**) MMP2/MMP9 gelatinolytic activities (as assayed by gelatin zymography) in MDA-MB-231 and shERβ MDA-MB-231 cells, before and after treatments (AG1478, E2, mix and 24 h). Each bar represents mean ± SD values from triplicate samples. Statistically significant differences are indicated accordingly: * *p* < 0.05, ** *p* < 0.01 and *** *p* < 0.01 compared to MDA-MB-231 control cells; ^+^ *p* < 0.05 and ^++^ *p* < 0.01 compared to shERβ MDA-MB-231 control cells; and ^#^ *p* < 0.05 and ^##^ *p* < 0.01 compared to E2-treated cells.

As depicted in Figure 1A, in MDA-MB-231 cells, inhibition of EGFR by AG1478 significantly downregulates the expression of both *MMP7* and *MT1-MMP* (ca. 25% and 60% respectively), while in the ERβ-suppressed cells no major differences were evident.

E2 treatment also causes statistically significant alterations in the ERβ positive MDA-MB-231 cells, as it reduces the expression of *MMP7* by 45% and *MT1-MMP* by 65%; however, in the shERβ MDA-MB-231 cells, the effects were not as prominent. Notably, in the Erβ-suppressed cells, dual treatment with AG1478 and E2 decreased the mRNA levels of *MMP7* by 80%.

Furthermore, since MMPs are proteolytic enzymes integral to the ECM degradation and remodeling process, it is important to study their activity levels. To that end, we performed gelatin zymography to assess the gelatinolytic activity of MMP9 and MMP2 (Figure 1B,C). Notably, treatment with AG1478 alone or in combination with E2 strongly reduces pro-MMP9, MMP9 and MMP2 activities in both cell lines.

### 3.2. EGFR/ERβ Axis Governs Metastasis-Related Functional Properties in Breast Cancer

The observed alterations in both expression and activity levels of the MMPs propelled us to investigate the impact of EGFR and ERβ in adhesion and invasion on collagen type I.

**Figure 2:**
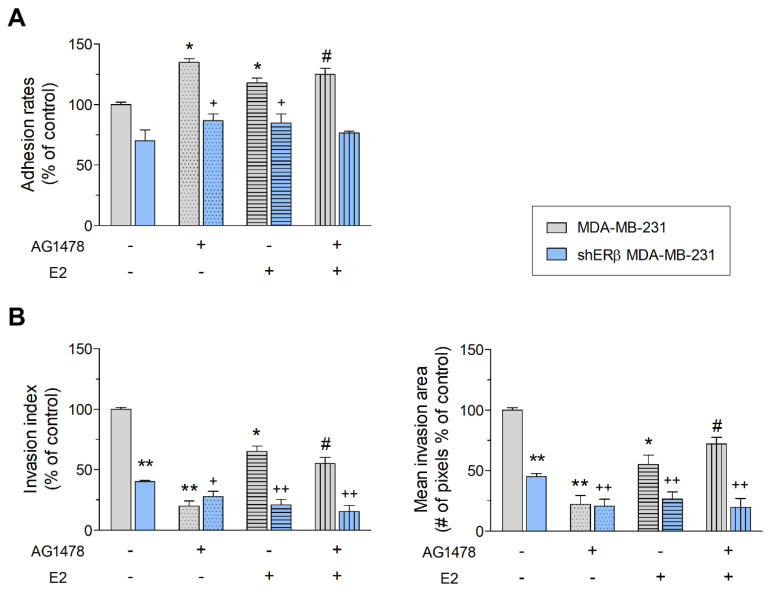
EGFR/ERβ axis regulates functional properties of MDA-MB-231 and shERβ MDA-MB-231 breast cancer cells. (**A**) Collagen type I adhesion. (**B**) Cell invasion in collagen type I matrix. Treatments with AG1478 (2 μM) and/or E2 (10 nM) were performed for 24 h prior in MDA-MB-231 and shERβ MDA-MB-231 cells. Each bar represents mean ± SD values from triplicate samples. Statistically significant differences are indicated accordingly: * *p* < 0.05 and ** *p* < 0.01 compared to MDA-MB-231 control cells; ^+^ *p* < 0.05 and ^++^ *p* < 0.01 compared to shERβ MDA-MB-231 control cells; and ^#^ *p* < 0.05 compared to E2-treated cells.

As depicted in Figure 2A, the inhibition of EGFR significantly induces the adhesion on collagen type I coating in both cell lines, as compared to respective control cells, hence enabling stronger attachment to the microenvironment.

Moreover, treated and untreated cells were seeded on collagen type I matrix, to compare their invasive capability. As illustrated in Figure 2B, ERβ suppression strongly downregulates invasion of MDA-MB-231 breast cancer cells in collagen type I matrix. Furthermore, EGFR inhibition leads to a reduction of the number and the area that breast cancer cells invade both in MDA-MB-231 (ca. 80%) and shERβ MDA-MB-231 cells (ca. 55%). Finally, addition of E2 also downregulates the invasion potential of both cell lines in a statistically significant manner, more so when combined with EGFR inhibition in shERβ MDA-MB-231 cells.

### 3.3. Morphology of Breast Cancer Cells Cultured on Millipore Filter Covered by Type I Collagen (200 µg/mL)

Initially, untreated (control) and treated (E2, AG1478 and mix) MDA-MB-231 and shERβ MDA-MB-231 cells cultivated on a Millipore filter covered by type I collagen 200 µg/mL were analyzed at SEM. In all of these groups, the collagen coating appeared as a thin layer of single fibrils not evenly spread. Nevertheless, almost all of Millipore filter holes were completely closed and masked by collagen fibrils, which strongly adhered to the Millipore filter.

**Figure 3:**
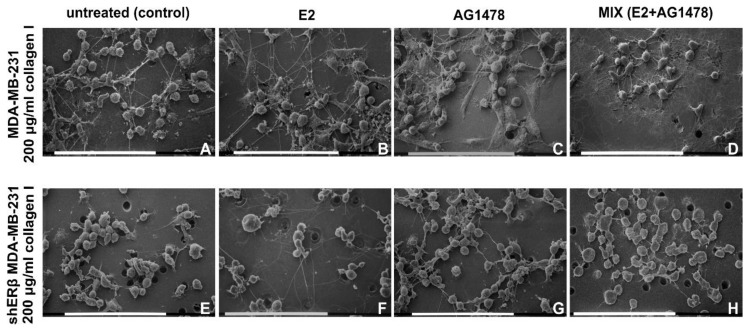
MDA-MB-231 and shERβ MDA-MB-231 cells cultured on Millipore filter covered by 200 µg/mL type I collagen. (**A**) Untreated MDA-MB-231 globular-shaped cells are grouped in clusters of a few cells showing long, thin filopodia and tunneling nanotubes (TNTs). (**B**) Most of MDA-MB-231 cells treated with E2 show a globular shape, but elongated-fusiform and flattened-polygonal cells are also observable. (**C**) Thin long filopodia are present similarly to untreated cells, but TNTs seem more numerous vs. control group MDA-MB-231 cells treated with AG1478 include globular, elongated-fusiform but a higher number of flattened-polygonal-shaped cells vs. control and E2 treated groups. Thin, long filopodia and TNTs are still detectable. (**D**) After both E2 and AG1478 treatment (mix group) MDA-MB-231 cells show globular, flattened-polygonal and few elongated-fusiform phenotypes. TNTs and many long, thin filopodia adhering to the collagen substrate and radially arising from cells give the cells a “spider” aspect. (**E**) Untreated shERβ MDA-MB-231 mainly includes smooth globular cells grouped in tight contact and showing short thin filopodia and few TNTs. (**F**) The same cells treated with E2 still exhibit a globular shape but appear in isolated groups of two or three cells showing longer filopodia vs. untreated control group. (**G**) After AG1478 treatment, the shERβ MDA-MB-231 globular-shaped cells again look grouped and show more contact one to each other. Fewer short and thin filopodia and intercellular TNTs are present. (**H**) After the combined treatment of E2 and AG1478, shERβ MDA-MB-231 cells look relatively grouped, displaying the same globular shape and show few short filopodia and TNTs. Bar = 0.1 mm.

MDA-MB-231 control cells mostly showed a globular shaped phenotype with only few flattened-polygonal- and elongated-fusiform-shaped cells; all cells presented extracellular microvesicles (EVs) and microvilli on their cytoplasmic surface, and some of them developed many long, thin filopodia (30–80 µm in length and 300–500 nm in diameter). The globular cells appeared partially grouped in clusters of few cells often connected one another by straight and very thin (50–300 nm) bridge-like intercellular cytoplasmic processes. They were distinguishable from the collagen fibrils, as they were positioned above and suspended from the culture ground, whereas collagen fibrils strongly adhered to the Millipore surface. These intercellular cytoplasmic connections were morphologically comparable to previously described tunneling nanotubes (TNTs) in 3D breast cancer cell cultures [37] (Figure 3A).

The addition of exogenous E2 favored a globular shape, although elongated-fusiform and few flattened-polygonal cells were also present. Microvilli, EVs and thin, long filopodia were visible, similar to untreated cells, but TNTs were more abundant compared to the control group (Figure 3B). MDA-MB-231 cells treated with AG1478, which allosterically inhibits EGFR, developed microvilli, EVs, thin, long filopodia and TNTs and included the same cell phenotypes previously described; however, the flattened-polygonal cells were present in larger numbers than in control samples (Figure 3C). Combined E2 and AG1478 treatment (mix group) developed globular, flattened-polygonal and few elongated-fusiform phenotypes. Microvilli, EVs, TNTs and many long, thin filopodia adhering to the collagen substrate radially spread from each cell, thus giving them a “spider” aspect (Figure 3D).

Control shERβ MDA-MB-231 cells cultivated on low concentration type I collagen fibrils were mainly globular shaped with few flattened-polygonal ones, but they looked smoother than the MDA-MB-231 cells and grouped in tight contact. Fewer microvilli, EVs, short, thin filopodia and few TNTs were also detectable (Figure 3E). When treated with E2, shERβ cells mainly exhibited a globular shape with few EVs, whilst growing isolated in groups of two or three cells and showing longer filopodia when compared to untreated control group (Figure 3F). After AG1478 treatment the shERβ, MDA-MB-231 globular cells appeared more grouped with increased cell–cell contacts. Microvilli and EVs seemed to be reduced by the AG1478 treatment, whereas short, thin filopodia and few intercellular TNTs were still detectable (Figure 3G). In the mix group, the same globular shaped cells were relatively grouped and showed few EVs, but also few short filopodia and TNTs (Figure 3H).

### 3.4. Morphology of Breast Cancer Cells Cultured on Millipore Filter Covered by Type I Collagen (3000 µg/mL)

Untreated (control) and treated (E2, AG1478 and mix) MDA-MB-231 and shERβ MDA-MB-231 cells cultivated on a Millipore filter covered by type I collagen 3000 µg/mL were observed at SEM. In all of these groups, the collagen coating and the Millipore holes were not easily observable. In fact, in most of the groups, the cells highly proliferated in different sheets that completely covered a uniform relatively thick layer of densely arranged fibrils adhering to the Millipore filter.

**Figure 4:**
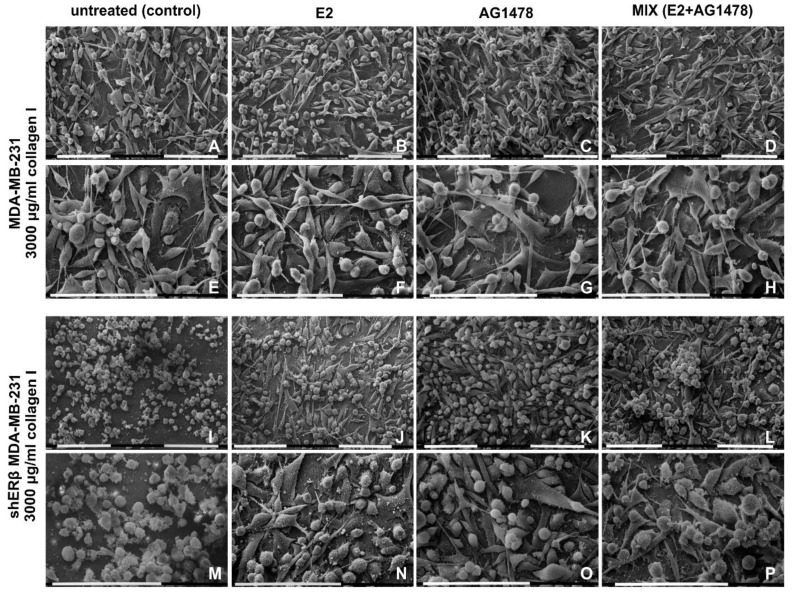
MDA-MB-231 and shERβ MDA-MB-231 cells cultured on Millipore filter covered by 3000 µg/mL type I collagen. (**A**,**E**) Untreated MDA-MB-231 cells include globular and elongated-fusiform phenotypes growing on flattened-polygonal cells strongly adhering to the collagen layer. Intercellular TNTs and long filopodia mainly originating from elongated-fusiform cells are visible. (**B**,**F**) When compared to the untreated samples, the same cells treated with E2 show a slight increase of globular and elongated-fusiform cells on flattened-polygonal ones adhering to the collagen substrate. Horizontal long conical filopodia and short intercellular TNTs are present. (**C**,**G**) MDA-MB-231 cells treated with AG1478 still exhibited globular and elongated-fusiform phenotypes on slightly increased flattened-polygonal cells. Long filopodia and TNTs are visible. (**D**,**H**) After a mix (E2 and AG1478) treatment, the MDA-MB-231 cells show a layer of flattened-polygonal cells adhering to the collagen substrate and covered by slightly decreased globular and elongated-fusiform cells with both long filopodia and TNTs. (**I**,**M**) Untreated shERβ MDA-MB-231 cells mainly include a globular cell phenotype. Cells appear mainly grouped and are occasionally connected by short intercellular TNTs; no filopodia are detectable. (**J**,**N**) When the same cells were treated with E2, mainly isolated globular and elongated-fusiform cells grew on a layer of flattened-polygonal cells. Cells developed EVs and microvilli, long filopodia and TNTs vs. control group. (**K**,**O**) shERβ MDA-MB-231 cells treated with AG1478 include many globular cells and few elongated-fusiform cells, all covering the flattened-polygonal ones. Cells are in very tight contact with each other, are uniformly distributed and show a smoother surface with few microvilli and fewer EVs, if compared to control and E2 groups. Long filopodia and TNTs were detectable. (**L**,**P**) After both E2 and AG 1478 treatments (mix group), shERB MDA-MB-231 cells included few elongated-fusiform- and many globular-shaped cells growing grouped in more than one layer on the flattened-polygonal ones. Filopodia are detectable. Bar = 0.1 mm.

**Figure 5:**
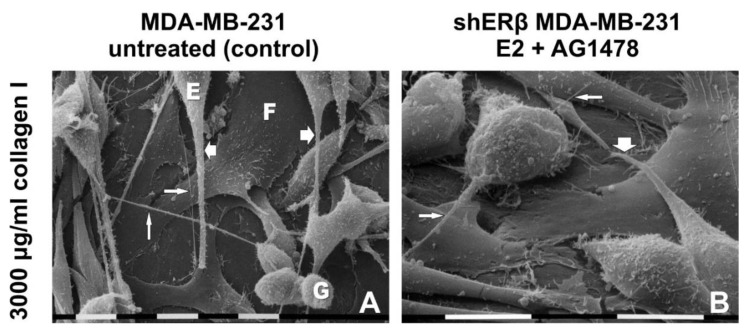
(**A**) Untreated MDA-MB-231 cells cultivated on a Millipore filter coated by 3000 µg/mL type I collagen: Flattened-polygonal cells (F) adhering to the collagen substrate are covered by globular (G) and elongated-fusiform (E) cells exhibiting microvilli and EVs. Long conical-shaped filopodia (large arrows) showing microvilli and EVs on their surface originate from elongated-fusiform cells. Straight intercellular TNTs (narrow arrow) are also present. Bar = 10 µm. (**B**) shERβ MDA-MB-231 cells treated with both E2 and AG1478 (mix) and cultivated on a Millipore filter coated by 3.0 mg/mL type I collagen: Globular and elongated cells showing microvilli on the cytoplasmic surface grow on flattened-polygonal cells just adhering to the collagen layer. A conical filopodia (large arrow) develops from an elongated-fusiform cell. Short straight intercellular TNTs (narrow arrows) are detectable. Bar = 10 µm.

Control MDA-MB-231 cells showed three different cell phenotypes: globular, elongated-fusiform and flattened-polygonal. The latter strongly adhered to the collagen layer and were almost completely covered by the globular and the elongated-fusiform ones. EVs on the cytoplasmic surface were more present in globular and elongated-superficial fusiform cells. These two cell phenotypes showed intercellular TNTs (30–50 nm in diameter); some of them exhibited few and isolated rounded cytoplasmic protrusions similar to EVs on their surface. Long filopodia (20–80 µm in length and 0.3–2 µm in diameter) mainly originating from elongated-fusiform cells showed many microvilli and EVs on their cytoplasmic surface (Figure 4A,E and Figure 5A).

E2 addition gave rise to flattened-polygonal cells adhering to the collagen substrate. Similar to the untreated group, globular and elongated-fusiform cells with microvilli and EVs grew above the flattened-polygonal ones, but a slight increase of the globular and elongated-fusiform ones versus the control untreated samples could be observed. Long conical filopodia (20–80 µm in length) with many EVs along with microvilli and intercellular TNTs with EVs on their surface were also visible (Figure 4B,F). After EGFR inhibition, MDA-MB-231 cells still exhibited globular, elongated-fusiform phenotypes showing microvilli and few EVs while growing on flattened-polygonal cells. However, a slight reduction of the elongated cells and an increase of the polygonal ones compared to the control group were noted. Filopodia with microvilli and few EVs on their surface and TNTs associated to EVs were also visible similarly to the previous groups (Figure 4C,G). After a mix treatment, the MDA-MB-231 cells showed a layer of flattened-polygonal cells adhering to the collagen substrate and covered by slightly decreased globular and elongated-fusiform cells. EVs, microvilli, long filopodia and TNTs were also present (Figure 4D,H).

Untreated shERβ MDA-MB-231 cells mainly included a globular cell phenotype when cultivated in higher collagen type I concentration. Cells appeared mainly grouped and showed fewer EVs and microvilli if compared to untreated MDA-MB-231 cells. Short intercellular TNTs (10–40 µm) were sometimes connecting the globular cells, whereas no filopodia were detectable (Figure 4I,M). When treated with E2, shERβ MDA-MB-231 cells presented globular and elongated-fusiform phenotypes, whereas growing on a layer of flattened-polygonal cells. Furthermore, cells developed more evident EVs and microvilli, long filopodia and TNTs with EVs on their surface than in the control group (Figure 4J,N). The same cells treated with AG1478 still included many globular cells but fewer elongated-fusiform cells, all covering the flattened-polygonal ones. Cells appeared in very tight contact, uniformly distributed on the flattened ones and showed a smoother surface with few microvilli and fewer EVs if compared to control and E2 groups. Long filopodia and TNTs were detectable (Figure 4K,O and Figure 5B). After dual E2 and AG1478 treatment, shERβ MDA-MB-231 cells included few elongated-fusiform and many globular-shaped cells growing grouped in more than one layer on top of the flattened-polygonal cells. Microvilli and EVs, TNTs, and filopodia were also present (Figure 4L,P).

### 3.5. Morphology of Breast Cancer Cells Cultured on Millipore Filter Covered by FN

Apart from collagen type I, FN is a main ECM substrate known to play crucial roles in cell adhesion [42]. Therefore, we further examined the effects of FN on cell morphology and aggressiveness of both cell lines using SEM.

Untreated (control) and treated (E2, AG1478 and mix) MDA-MB-231 and shERβ MDA-MB-231 cells cultivated on a Millipore filter covered by FN 130 µg/mL were observed at SEM. In most areas of most samples, the cells proliferated in different layers covering both FN and Millipore filter. When the Millipore filter surface was not covered by cells, FN was not always clearly detectable but was able to fill almost all the Millipore holes.

**Figure 6:**
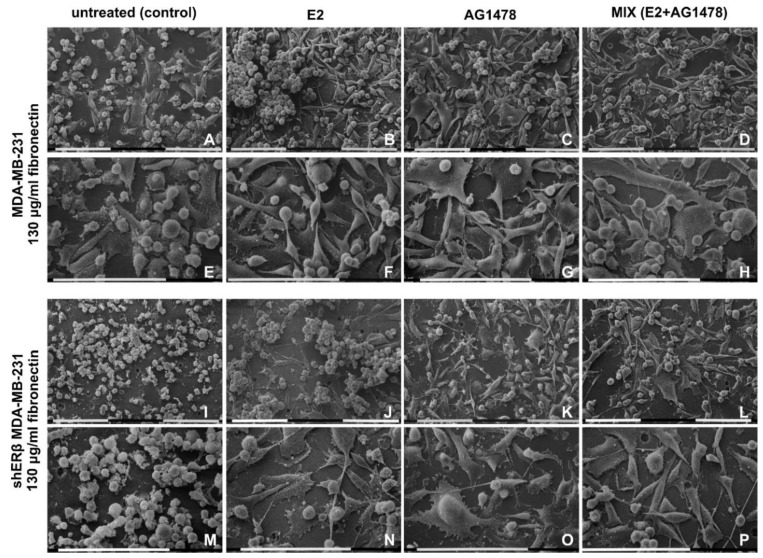
MDA-MB-231 and shERβ MDA-MB-231 cells cultured on Millipore filter covered by 130 µg/mL FN. (**A**,**E**) Untreated MDA-MB-231 cells displayed three different cell phenotypes: more numerous globular cells, large flattened-polygonal cells and fewer elongated-fusiform ones. Very few short filopodia and only few intercellular TNTs are observable. (**B**,**F**) With E2 treatment, MDA-MB-231 cells show isolated or grouped globular cells usually growing in more than one layer on more numerous elongated-fusiform and flattened-polygonal ones. Long conical filopodia and intercellular TNTs are visible. (**C**,**G**) After treatment with AG1478, MDA-MB-231 cells appear more grouped and include globular phenotypes growing on fewer elongated-fusiform and increased very large flattened-polygonal ones vs. control group. Few filopodia and TNTs are also visible. (**D**,**H**) With mix treatment, MDA-MB-231 cells appear grouped and show globular, large flattened-polygonal and elongated-fusiform shapes; long filopodia and TNTs. (**I**,**M**) Untreated shERβ MDA-MB-231 cells mainly show grouped globular cells but some isolated globular cells with few microvilli, and few EVs are also present. No filopodia are observable, whereas few short TNTs connect adjacent cells. (**J**,**N**) After E2 treatment, shERβ MDA-MB-231 cells display isolated and grouped globular cells growing in different layers on flattened-polygonal and isolated elongated-fusiform cells. Cells display microvilli and few EVs, long filopodia and TNTs. (**K**,**O**) After AG1478 treatment, the shERβ MDA-MB-231 cells include many large flattened-polygonal, globular and elongated-fusiform cells which appeared a little more grouped when compared to the E2 group. Few microvilli and few EVs, long filopodia and TNTs are observable. (**L**,**P**) The same cells treated with both E2 and AG1478 (mix) comprise globular, flattened-polygonal and elongated-fusiform cells equally represented and exhibiting few EVs and few microvilli, long filopodia and TNTs. Bar = 0.1 mm.

Untreated MDA-MB-231 cells displayed three different cell phenotypes: numerous globular cells, large flattened-polygonal cells sometimes in tight contact and isolated fewer elongated-fusiform ones were recognizable. Microvilli and EVs were evident on the cytoplasmic surface; very few short filopodia and only a few intercellular TNTs (30–50 nm in diameter) were observed (Figure 6A,E).

**Figure 7:**
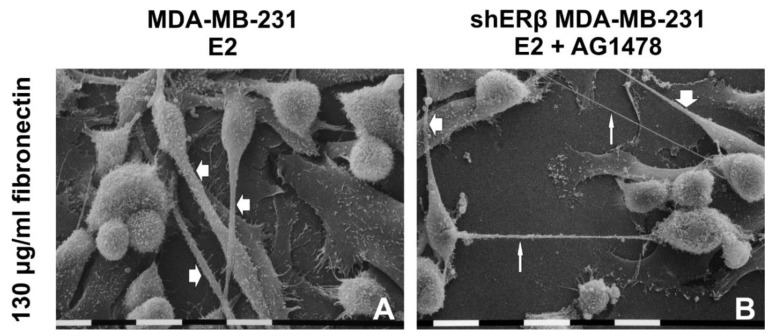
(**A**) MDA-MB-231 cells treated with E2 and cultivated on a Millipore filter coated by 130 µg/mL FN: Flattened-polygonal cells adhering to the collagen substrate are covered by globular and elongated-fusiform cells showing microvilli and EVs. Long conical-shaped filopodia (large arrows) including microvilli and EVs on their surface arise from elongated-fusiform cells. Bar = 10 µm. (**B**) shERβ MDA-MB-231 cells treated with both E2 and AG1478 (mix) and cultivated on a Millipore filter coated by 130 µg/mL FN: Globular and elongated-fusiform cells on the Millipore filter are detectable. Long filopodia (large arrows) developing from elongate cells and straight intercellular TNTs (narrow arrows) with EVs on their surface are visible. Bar = 10 µm.

E2 treatment on MDA-MB-231 cells leads to an increase of isolated or grouped globular cells usually growing in more than one layer on top of flattened-polygonal and elongated-fusiform cells, with the latter being more present than in the control group. All the cells showed microvilli, EVs and long conical filopodia (20–80 µm in length) including many EVs and microvilli on their surface and intercellular TNTs (Figure 6B,F and Figure 7A).

MDA-MB-231 cells treated with AG1478 appeared growing on elongated-fusiform and very large flattened-polygonal cells; the cells were more grouped than the control ones but still included globular cells showing microvilli and few EVs. Additionally, there was a slight reduction of the elongated cells and an increase of the polygonal ones versus the control group, while few filopodia with microvilli or EVs on their surface and TNTs associated to EVs were also visible (Figure 6C,G). Mix treatment with E2 and AG1478 contributed to grouped growing cells with globular shaped phenotype, as well as large flattened-polygonal and elongated-fusiform cells. Moreover, EVs, microvilli, few long filopodia and TNTs were also present (Figure 6D,H).

shERβ MDA-MB-231 cells cultivated on FN mainly included a globular cell phenotype, grouped, with cell-cell contacts even though isolated globular cells, few flattened-polygonal and elongated-fusiform cells were also present. Few microvilli and EVs on cell surface and almost no filopodia were detectable while few short TNTs connected adjacent cells (Figure 6I,M). When shERβ MDA-MB-231 cells were treated with E2 isolated and grouped globular cells grew in different layers on flattened-polygonal and isolated elongated-fusiform cells. Microvilli and EVs were recognizable on cell surface and long filopodia and TNTs with EVs on their surface were detectable (Figure 6J,N). After EGFR inhibition, shERβ MDA-MB-231 cell culture included many large flattened-polygonal, globular and elongated-fusiform cells, which appeared a little more grouped when compared to the E2 group. Few EVs, few microvilli, long filopodia and TNTs were observed (Figure 6K,O). Finally, shERβ MDA-MB-231 cells treated with both E2 and AG1478 included globular, flattened-polygonal and elongated-fusiform cells equally represented and exhibited few EVs and few microvilli, long filopodia and TNTs. (Figure 6L,P and Figure 7B).

## 4. Discussion

Tumor microenvironment, particularly ECM, is recognized as a critical regulator of breast cancer cell behavior. Matrix macromolecules, especially those implicated in ECM degradation and remodeling, like MMPs, can impact various functional cell properties hence advancing EMT [43]. Cancer cell populations demonstrate significant heterogeneity, which explains the indirect link between EMT and metastasis, that only some cells from the primary tumor will undergo EMT and metastasize distal tissues [44,45]. However, for a cancer cell to be able to metastasize, it needs to undergo EMT-related changes. Specifically, in vitro studies in breast cancer, lung adenocarcinoma and ovarian cancer cells show cancer cells in different EMT states which exhibit increased invasion and migration [46,47,48]. Similarly, studies in xenograft models and primary cancers associate EMT with tumor cell dissemination, poor survival and resistance to therapy in several cases, including pancreatic, ovarian, lung and breast cancer [49,50,51]. MMPs contribute to cancer progression and tumor growth by activating growth factors, as well as their receptors and promoting angiogenesis and invasion. Besides, studies in breast cancer patients correlate MMP deregulation with mammary tumor development, increased metastatic potential and worst survival prognosis [52].

The association between MMPs and growth factor receptors’ activation prompted as to evaluate the role of EGFR in invasion and metastasis. In breast cancer the EGFR/ERs crosstalk is crucial in regulating MMPs expression and functional cell properties [53]. Notably, when ERβ is suppressed in MDA-MB-231 cells, EGFR expression is downregulated (ca. 50%). Still, its downstream signaling can impact several other pathways. Hence, this study focused on the evaluation of EGFR inhibition in TNBC cells of different ERβ status. As shown in the results, EGFR inhibition downregulates the expression and activity levels of MMP2, -7, -9 and MT1-MMP, whereas ERβ seemingly mediates *MMP7* and *MT1-MMP* expression. These results were in agreement with the respectively increased adhesion and reduced invasion levels when AG1478 and/or E2-treated cells were cultivated on collagen type I, thus proving the connection between EGFR activation and ECM remodeling that leads to EMT advancement.

Standard 2D cell cultures display significant limitations in evaluating the morphology and, thus, the behavior of breast cancer cells, since they do not allow a natural cell growth [54]. Three-dimensional scaffolds containing natural structural components of the tumor microenvironment, such as collagen I and FN, are preferred, as these matrices can better simulate the tumor microenvironment and mimic the in vivo conditions that allow breast cancer cells to grow and interact, both with one another and with the surrounding microenvironment [55,56,57]. Interestingly, collagen and FN influence cancer cell migration and behavior in an opposite way. Mechanical extension of FN fibers seems to decrease epithelial cell adhesion and migration [58], whereas, on the contrary, aligned straight collagen fibers can favor breast cancer migration and invasion [59,60,61]. Cell-matrix adhesion is mainly facilitated by integrins that bind to various ECM proteins (FN, collagen I, laminin, etc.) [62]. Integrin heterodimers bind different matrix proteins to activate intracellular signaling pathways; hence, depending on the integrin dimer, different pathways will be induced every time [63]. Common FN-binding integrins include αvβ5, αvb1, α5β1 and αvb3, whereas α9β1, α1β1, α4β1 and α2β1 bind collagen [64,65,66]. These differences between collagen and FN explain the different and opposite effects that these substrates evoke on breast cancer cells.

Based on previously published results [37,67] and the confirmed interplay between EGFR and ERs, our goal for this study was to evaluate the impact that different matrix substrates have on morphological aspects of breast cancer cells, following EGFR inhibition in the presence and absence of 17β-estradiol. The use of two different types of matrices provided us with the opportunity to underscore the impact the matrix composition can have in cell shape and morphological characteristics. In addition, the two vastly different collagen type I concentrations (200 and 3000 µg/mL) helped to better highlight the impact of thicker substrate in breast cancer cell behavior. Overall, we demonstrated that aggressive MDA-MB-231 breast cancer cells cultivated on a matrix scaffold of FN clearly develop less EVs, long filopodia and TNTs. This may be correlated with the functional role of integrins (e.g., α5β1) that can bind FN [64] and, due to mechanosensing, promote conformational changes in other proteins of adhesomes like talin, paxillin and p130Cas, thus modulating cell adhesion [68,69,70]. On the other hand, we observed that, in the presence of collagen type I as a culture scaffold, the aggressive morphology of MDA-MB-231 cells is conserved, especially in the denser substrate cultures; when the concentration of collagen type I was increased to 3000 µg/mL, the MDA-MB-231 control cell morphology partially shifted from a mainly globular shape in the 200 µg/mL cultures to spindle-like cells, but also flattened polygonal ones, thus demonstrating the impact of a thicker collagen substrate with denser fibrils and different porosity. This observation is in agreement with the literature, as, even though the mechanisms behind 3D adhesion mechanosensing are not fully elucidated, it is known that lower concentrations of collagen type I resemble the normal ECM composition of the human body, while increased matrix stiffness and density are associated with cancer progression [71,72,73]. Concerning the shERβ MDA-MB-231 cancer cells, we demonstrated that, regardless of the substrate (collagen type I or FN), the untreated cultures mainly include globular cells.

E2 addition reduced cell–cell contacts in the 200 μg/mL type I collagen scaffold and increased adhesion in FN, hence advancing the aggressive phenotype of breast cancer cells. Moreover, E2 actions favor the development formation of long, thin filopodia and TNTs in every substrate we tested and in both cell lines. Very long filopodia are thought to have a sensory and mechanical role during cell migration. In addition, they facilitate exosomal capture, uptake and entry in the cell [37,74]. Direct intercellular cytoplasmic connections, like TNTs, develop when traditional intercellular communication via cell junctions is not available, mainly due to cells being isolated [75]. TNTs are related to intercellular molecular exchange, including exosomes and mitochondria, and, therefore, they could be the expression of enhanced cell–cell interplay [76,77,78]. On the other hand, EGFR inhibition induced cell contacts and grouped cell growth, while it bestowed less aggressive morphological traits on the cells, including a flat polygonal or globular shape and less EVs and TNTs. The presence of E2 on top of EGFR inhibition did not attribute to great morphological alterations compared to AG1478 monotreatment, therefore suggesting the need for EGFR activation for E2 to exert its actions.

## 5. Conclusions

Collectively, this study explored the connection between ERβ, EGFR and ECM. The obtained results highlight the importance of ERβ/EGFR crosstalk in the modulation of critical cell functional properties (i.e., invasion and adhesion), as well as the mRNA expression and gelatinolytic activity of matrix macromolecules (MMPs) involved in ECM remodeling and cancer progression. The obtained data pinpoint the importance of matrix substrates (collagen type I and FN) in the regulation of the mesenchymal morphology of MDA-MB-231 breast cancer cells. Furthermore, we confirmed that ERβ suppression leads to less filopodia and TNTs, therefore implying a synergistic effect of ERβ suppression and differentiated matrix composition in the preservation of a less aggressive phenotype in breast cancer, cells in accordance with the expression profiles of MMPs. In addition, EGFR inhibition and the presence of E2 resulted in important morphological changes both in MDA-MB-231 and shERβ MDA-MB-231 cells, suggesting the significance of EGFR signaling in the modulation of breast cancer cell morphological characteristics. Further investigation, including mechanistic aspects, will contribute to a better understanding of the underlying mechanisms of breast cancer cell aggressiveness, thus improving the therapeutic approaches for breast cancer management.

## Figures and Tables

**Table 1:** Primer sequences used for quantitative RT-PCR.

Gene		Primer Sequence (5′-3′)	T_annealing_ (°C)
**MMP7**	F	GCTGGCTCATGCCTTTGC	60
	R	TCCTCATCGAAGTGAGCATCTC	
**MT1-MMP**	F	CATGGGCAGCGATGAAGTCT	60
	R	CCAGTATTTGTTCCCCTTGTAGAAGTA	
**ACTB**	F	TCAAGATCATTGCTCCTCCTGAG	60
	R	ACATCTGCTGGAAGGTGGACA	
